# Regional increases of cortical thickness in untreated, first-episode major depressive disorder

**DOI:** 10.1038/tp.2014.18

**Published:** 2014-04-08

**Authors:** L Qiu, S Lui, W Kuang, X Huang, J Li, J Li, J Zhang, H Chen, J A Sweeney, Q Gong

**Affiliations:** 1 Huaxi MR Research Center (HMRRC), Department of Radiology, West China Hospital of Sichuan University, Chengdu, China; 2 Department of Radiology, The Second People's Hospital of Yibin, Yibin, China; 3 Department of Psychiatry, The State Key Lab of Biotherapy, West China Hospital of Sichuan University, Chengdu, China; 4 Key laboratory for Neuroinformation of the Ministry of Education, School of Life Science and Technology, University of Electronic Science and Technology of China, Chengdu, China; 5 Department of Psychiatry, University of Texas Southwestern, Dallas, TX, USA; 6 Department of Pediatrics, University of Texas Southwestern, Dallas, TX, USA

## Abstract

The large majority of structural MRI studies of major depressive disorder (MDD) investigated volumetric changes in chronic medicated patients in whom course of illness and treatment effects may impact anatomic measurements. Further, in few studies, separate measurements of cortical thickness and surface area have been performed that reflect different neurobiological processes regulated by different genetic mechanisms. In the present study, we investigated both cortical thickness and surface area in first-episode, treatment-naïve, mid-life MDD to elucidate the core pathophysiology of this disease and its early impact on the brain. We observed increased cortical thickness in the right hemisphere, including medial orbitofrontal gyrus, pars opercularis, rostral middle frontal gyrus and supramarginal gyrus. Increased thickness of rostral middle frontal gyrus was negatively related with depression severity on the Hamilton Depression Rating Scale. Furthermore, MDD patients showed significantly increased associations in cortical thickness measurements among areas where increased cortical thickness was observed. Analysis of pial area revealed a trend toward increased surface area in the left parahippocampal gyrus in MDD. To permit comparison of our data with those of previous gray matter volume studies, voxel-based morphometry was performed. That analysis revealed significantly increased gray matter volume in left paracentral lobule, left superior frontal gyrus, bilateral cuneus and thalamus which form limbic-cortico–striato–pallido–thalamic loops. These changes in first-episode, treatment-naïve, mid-life MDD patients may reflect an active illness-related cortical change close to illness onset, and thus potentially provide important new insight into the early neurobiology of the disorder.

## Introduction

Though major depressive disorder (MDD) ranks as the leading cause of years lived with disability among all diseases,^1^ the pathogenesis and pathophysiological processes of this illness are not well understood. Investigations of brain morphology may help to clarify the impact of this disorder on the brain. Most previous structural studies of brain anatomy in MDD investigated volumetric changes in chronic medicated patients. These studies most commonly reported reduced gray matter volume in regions including anterior cingulate,^2,3^ orbitofrontal cortex,^2, 3, 4^ dorsal anterolateral and ventrolateral prefrontal cortex, precuneus and inferior parietal lobule.^3^ Two meta-analyses reported smaller volumes of the basal ganglia, thalamus, hippocampus, anterior cingulate and orbitofrontal cortex in MDD.^5,6^ In addition, three studies^7, 8, 9^ measured cortical thickness in elderly MDD patients and one of these studies^9^ found significantly decreased cortical thickness. Other studies^10, 11, 12^ reported reduced cortical thickness in depressed patients with type 2 diabetes patients^10^ and MDD with high risk for suicide^11^ when compared with healthy controls and depressed patients at lower risk for suicide respectively. Another study found significantly thinner posterior cingulate cortex in non-remitters with MDD than in remitters.^12^

Although the majority of studies reported reduced cortical volumes, some studies have reported volume increases in precuneus, cingulate gyrus, middle frontal gyrus and angular gyrus in adult patients.^13, 14, 15^ Pediatric MDD patients early in the course of illness and with positive family history have been reported to have significantly larger left prefrontal cortical volumes than those with a negative family history and controls.^16^ This inconsistency regarding reports of increased and decreased regional volumes in MDD is potentially due to factors such as field strength of the MR scanner, co-morbid illness, heterogeneity and chronicity of patients, medication effects, as well as factors relating to unique aspects of childhood and late-onset depression.

Few studies have examined anatomical changes in first-episode, drug-naïve adult MDD patients which may be important for elucidating the core pathophysiology of this illness independent from these potential confounds and for evaluating the trajectory of its early impact on the brain in longitudinal studies.^17,18^ In particular, one recent study by van Eijndhoven *et al.*^19^ investigated cortical thickness in first episode depression by studying 20 medication-naive currently depressed patients and 20 medication-free recovered MDD patients. They reported increased cortical thickness in the temporal pole, the caudal anterior and posterior cingulate cortex, and rostral dorsolateral prefrontal cortex (DLPFC) in the first episode medication-free patient group, and reduced cortical thickness in the medial orbitofrontal gyrus. Notably in that study, analysis was performed on the combined data of both the medication-naïve currently depressed patients and the medication-free recovered MDD patients relative to controls. Thus the results may be influenced by prior treatment.^19^

In addition to those factors, cortical gray matter volume measures combine influences of cortical thickness and surface area, features which are believed to be influenced by different genetic factors regulating sulcal patterning and the thickness of the cortical mantle itself.^20^ Previous studies^21, 22, 23^ have found that cortical volume is driven mostly by cortical surface area rather than cortical thickness. Advanced automated analytic techniques such as FreeSurfer (http://surfer.nmr.mgh.harvard.edu) now make possible the examination of cortical thickness and area *in vivo* using high spatial resolution structural MRI. Independent measurements of these aspects of cortical anatomy are important as cortical thickness reflects the size, density and arrangement of neurons, neuroglia and nerve fibers,^24^ and thus its measurement could provide important and relatively unique information about disease-specific neuroanatomical changes. For instance, the regional thinning of cortex can reflect reduced dendritic arborization or changing myelination at the gray/white matter interface^25^ within specific brain systems. Postmortem studies in MDD have reported modest thinning of the cortical ribbon and/or layer-specific decreases in cell density due to cell loss and cell atrophy in localized regions, including the rostral and caudal orbitofrontal and DLPFC.^26,27^ However, postmortem cytoarchitectural measurements have limitations, including tissue fixation artifacts and limited antemortem characterization, and they typically are done with chronic patients typically with a protracted course of illness.

In addition to the regional measurement of cortical thickness, correlational analysis of the pattern of cortical thickness^28, 29, 30, 31^ across the brain can provide information about changes in distributed brain circuitry. Regional changes of anatomical connection patterns of the human cerebral cortex are reflected in parallel changes in cortical thickness measurements as proposed by He *et al.*^32,33^ This has been examined in other neuropsychiatric disorders,^33, 34, 35^ but not yet in MDD. Correlational analysis of cortical thickness measurements from structural MRI data rests on the assumption that positive correlations indicate connectivity, as axonally connected regions are believed to have common trophic and maturational influences.^36,37^ In clinical studies, such correlational analyses thus have the potential to map networks that may undergo common pathological processes. Our previous studies of a smaller independent sample of treatment-naïve, mid-life MDD revealed both regional and functional connectivity alterations within cerebral circuitry.^38,39^ Thus, in the present study we hypothesized that alterations in regional cortical thickness and in the relationships of thickness patterns across the brain would exist within cortico-cortical circuits in first-episode, treatment-naïve, mid-life MDD patients.

## Materials and Methods

### Participants

Forty-six first-episode, drug-naïve, mid-life MDD patients and 46 healthy controls were recruited at the Department of Psychiatry at the West China Hospital of Sichuan University (Table 1). The diagnosis of depression was made using the SCID (Structured Clinical Interview for DSM Disorders) according to Diagnostic and Statistical Manual of Mental Disorders, 4th edition (DSM-IV) criteria. All patients had a score of at least 18 on the 17-item Hamilton Depression Rating Scale (HDRS). Duration of time from first illness manifestation to the time of the MRI scan ranged from 2–60 weeks. Potential participants were excluded if they had comorbid anxiety disorders, a history of seizures, head trauma, dementia, intellectual impairment, neurological disease or neurosurgery, substance abuse or dependence, chronic medical conditions, history of learning disabilities, psychotic symptoms or cardiovascular disease. Healthy controls had no personal history of psychiatric illness as determined by SCID-NP interviews, and no known psychiatric illness in first-degree relatives. No subject had previously received any psychotropic medications or psychotherapy. MDD patients and control subjects were matched in age, sex, handedness (all were right handed) and years of education (Table 1). The study was approved by the local research ethics committee, and all participants provided informed consent after receiving a full explanation of the purpose of the study and the study procedures.

### Data acquisition, processing and statistical analysis

MR scanning was carried out on a 3.0 T MR scanner (EXCITE, GE medical system, Milwaukee, WI, USA). Subjects were fitted with soft ear plugs, positioned comfortably in the coil and instructed to relax and remain still. Head motion was minimized with foam pads. High resolution three-dimensional T1-weighted images were acquired employing a spoiled gradient recalled sequence with TR/TE=8.5/3.4 ms, flip angle=12°, 156 axial slices with thickness=1 mm, axial FOV=24 × 24 cm^2^ and data matrix=256 × 256.

Constructions of cortical surface were developed from three-dimensional spoiled gradient recalled images using FreeSurfer software (http://surfer.nmr.mgh.harvard.edu/, vision 4.5.0), which uses automated surface reconstruction, transformation and high-resolution inter-subject alignment procedures to measure the thickness of the entire cortex.^40, 41, 42, 43, 44^ In brief, the procedure involves segmentation of white matter, tessellation of gray/white matter junctions, inflation of the folded surface tessellation patterns and automatic correction of topological defects in the resulting manifold. The thickness of each subject's cortical ribbon was computed on a uniform grid with 1 mm spacing across both cortical hemispheres, with thickness defined as the shortest distance between the gray/white and pial surface models,^40^ providing sub-millimeter resolution. Surface maps were generated following registration of all subjects' cortical reconstructions to a common average surface and then smoothed using a surface-based Gaussian kernel of 20 mm full width half-maximum.

Cortical thickness maps from patients and controls were compared using a general linear model with age and sex as covariates. As all images were aligned to a common surface template, we did not use intracranial volume as covariates. The regions that showed group differences in cortical thickness after FDR (false discovery rate) correction were extracted in common space for each individual. In exploratory analyses, partial correlations using age and sex as covariates were computed to examine relationships between the mean cortical thickness in regions with altered thickness and clinical characteristics, including duration (weeks) and severity of depression. Partial correlations of cortical thickness among brain regions with group differences were obtained for each subject group using age and sex as covariates. Testing for differences of correlation coefficients between patients and controls was performed using Snedecor's method,^45^ which uses Fisher's method to transform *r* values (correlational coefficients) to *z* values to test for significant differences in correlations between subject groups.

Analysis of pial area and volume were also performed. The analysis of pial area was vertex based using FreeSurfer software. The volume analysis was performed by using SPM8 software (http://www.fil.ion.ucl.ac.uk/spm) and the VBM8 (Voxel-Based Morphometry 8) toolbox (http://dbm.neuro.uni-jena.de/vbm8). Details of the analytic method are presented in Supplementary Materials.

## Results

### Alterations of cortical thickness

**Figure 1:**
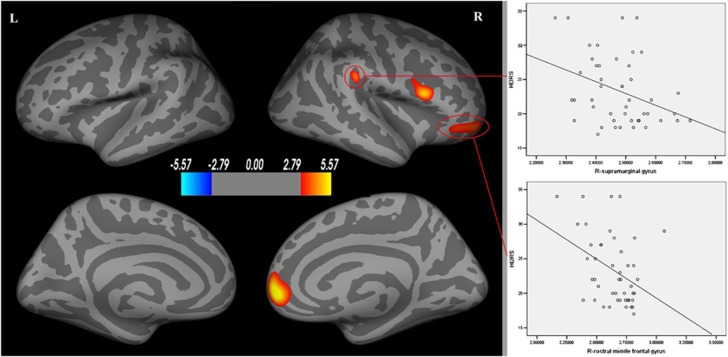
Areas with cortical thickness differences between healthy controls and patients with major depression (left) after FDR correction. Scatterplots show the negative correlation between HDRS with right rostral middle frontal gyrus and right supramarginal gyrus (right). Warmer colors (positive values) represent cortical thickening; cooler colors (negative values) represent significant cortical thinning in MDD patients. The color-coding for *P*-values is on a logarithmic scale of 1–6. L, left hemisphere; R, right hemisphere.

Compared with controls, MDD patients showed greater cortical thickness (*P*<0.05 after FDR correction) in right frontoparietal regions including medial orbitofrontal gyrus (Brodmann 9, 10, peak −log (*p*)=5.67), pars opercularis (Brodmann 44, peak −log (*p*)=4.81), rostral middle frontal gyrus (Brodmann 46, peak −log (*p*)=3.82) and supramarginal gyrus (Brodmann 40, peak −log (*p*)=3.98). No region with significantly decreased cortical thickness was found in MDD patients (Figure 1 and Table 2).

### Alterations of pial area and cortical volume

Analysis of pial area revealed a trend toward increased surface area in left parahippocampal gyrus in MDD (*P*<0.001 without correction). Detailed results of analyses with pial surface measurements are provided in Supplementary Materials.

Analyses of gray matter volume were performed by applying the VBM-DARTEL (Diffeomorphic Anatomical Registration using Exponentiated Lie algebra) analytic method. We observed increased volume in left paracentral lobule, left superior frontal gyrus, bilateral cuneus and thalamus after FDR correction in MDD patients.

### Relationship of cortical thickness with illness duration and depression severity

The increased cortical thickness of right rostral middle frontal gyrus was negatively correlated with HDRS scores (*r*=−0.34, *P*=0.020). A similar trend was observed between mean cortical thickness of right supramarginal gyrus and the HDRS (*r*=−0.29, *P*=0.051) (Figure 1 and Table 2). There was no significant correlation between illness duration and cortical thickness in regions with significantly increased thickness, though illness duration was typically brief in our patient group.

### Correlation of mean cortical thickness measurements across brain regions

**Figure 2:**
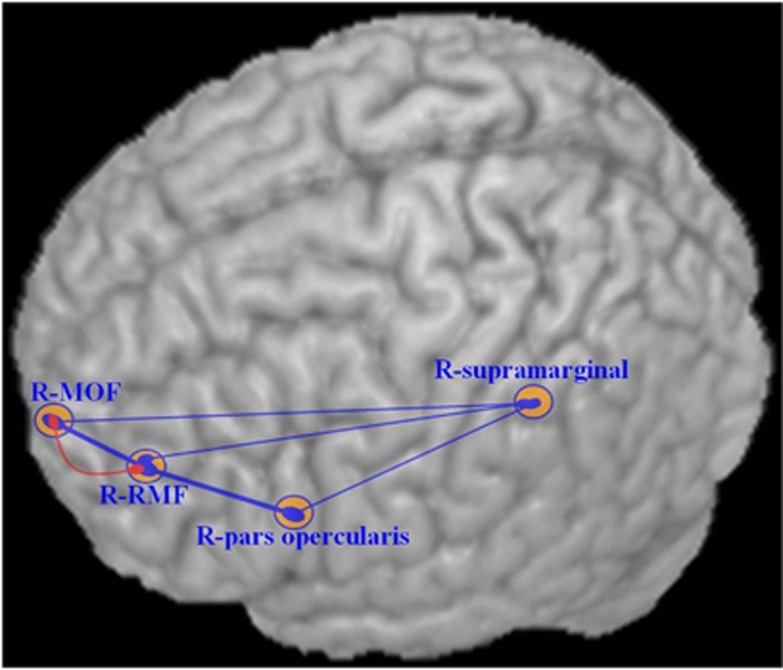
Correlations of mean cortical thickness across brain regions in MDD and healthy controls. The heavy line represents higher correlation coefficients (*r*>0.5, *P*≤0.001). The red line represents the significant positive correlations in healthy controls, and the blue line represents the significant positive correlations in MDD patients. MOF, medial orbitofrontal gyrus, R, right hemisphere, RMF, rostral middle frontal gyrus.

In MDD patients, all areas with increased cortical thickness showed significantly positive correlations with each other (*P*<0.05) suggesting a generalized effect of influences impacting regional cortical thickness. Controls showed no significant correlation among these areas except for a positive correlation between right medial orbitofrontal gyrus and right rostral middle frontal gyrus (*r*=0.35, *P*=0.019). Significantly greater correlation coefficients for interregional cortical thickness were found in patients relative to controls in all areas except for the associations of the right rostral middle frontal gyrus with right supramarginal gyrus and right medial orbitofrontal gyrus with right supramarginal gyrus (Figure 2 and Table 3).

## Discussion

To the best of our knowledge, the current study of cortical thickness is the largest study of brain morphometry in first-episode, treatment-naïve, mid-life MDD patients. It also is uncommon in analyzing cortical thickness, area and volumes. In stark contrast to observations of volume reduction in studies of chronic patients, we observed greater rather than reduced cortical thickness in early-course never-treated MDD patients in right medial orbitofrontal gyrus, right rostral middle frontal gyrus, right supramarginal gyrus and right pars opercularis. Correlations of cortical thickness measurements among these areas were significantly increased in MDD suggesting a broad coherent effect across several areas of association cortex. Modest negative correlations were found between cortical thickness and HDRS scores, indicating that the effect of increased thickness may be present in milder conditions or that it may represent a compensatory response to factors related to inflammation or other aspects of the pathophysiology of depression.

The most unique finding from our study is the observation of increased cortical thickness in early course untreated patients, which contrasts with multiple studies of chronic MDD patients showing reduced gray matter volume^2, 3, 4,15^ or decreased cortical thickness.^9^ The difference in our study findings from much of the existing literatures may be related to the fact that our patients were both treatment-naïve and very early in the course of the illness. Another recent study by van Eijndhoven *et al.*^19^ also reported increased cortical thickness in first-episode MDD in regions including the bilateral temporal poles, left posterior and rostral anterior cingulate cortex. In their study, only one brain region, left ventral-posterior medial orbital prefrontal cortex, showed reduced cortical thickness (Please see Figure 1 in reference 19).

Though the reasons for the increased thickness of neocortex in MDD are currently unclear, one possible account for this effect is that it may be related to an inflammatory response, representing a compensatory effect in the early stage of depression.^46^ In the early stage of inflammation, astrocytes, which constitute 90% cortical tissue volume, can be activated by the proinflammatory cytokines such as interleukin (IL)-6 and lead to cellular hypertrophy, astrocyte proliferation, process extension and interdigitation, which could increase cortical thickness.^47^ Furthermore, the activated astrocytes could promote neuronal survival by producing neurotrophic factors that promote recovery in central nervous system function. In other words, astrocyte activation could increase cortical thickness and produce increased levels of neurotrophic factors which could provide a neuronal protection effect and attenuate symptoms. Thus, the inverse relationship of increased thickness of right rostral middle frontal gyrus with lower HDRS scores in the MDD patients may represent a stronger neuronal protection effect by the activated astrocytes. Another study recently reported increased cortical thickness^48^ in right medial orbitofrontal cortex in individuals at increased risk for MDD, raising the possibility that the effect we observed close to illness onset may precede and be related to the pathophysiology of early illness manifestations. Illness-related physiological hyperfunction (higher metabolism and blood flow) also might through various mechanisms increase cortical thickness. These processes may be particularly relevant early in the course of illness, whereas the reduced gray matter volume^2, 3, 4,15^ and decreased cortical thickness^9^ in chronic MDD may be due to neurotoxic effect of recurrent or chronic depression that might occur over the longer course of illness progression.^49^ The exact mechanism for increased regional cortical thickness in MDD remains to be elucidated, hence further research is needed to clarify the cause, course and consequences of increased regional cortical thickness early in the course of MDD. Further, the extent to which the alterations we observed represent pre-existing risk factors or early effects of illness, and whether it is differentially seen in a subgroup of patients, remain to be determined.

The increased cortical thickness observed in the present study was primarily found in prefrontal cortex, suggesting a particularly important role of this brain region in MDD. This observation parallels previous volumetric studies of prefrontal cortex in MDD, especially of orbital and medial prefrontal regions and DLPFC, in which abnormalities have been widely reported.^2, 3, 4,14,50^ In contrast to our finding of increased cortical thickness in the right (rostral-superior) medial orbitofrontal gyrus, van Eijndhoven *et al.*^19^ reported reduced thickness in left (ventral-posterior) medial orbitofrontal cortex. Although the areas are somewhat different, van Eijndhoven *et al.*^19^ combined data of 20 medication-naive and 20 medication-free recovered MDD patients in their analyses, so that previous treatment might account for some differences between our studies. Discrepancies between these study findings might also be attributed to factors such as differences in sample size, subject handedness, ethnic background and illness duration. Notably, the illness duration on average is 17.4 weeks in present study, but 28.4 weeks on average for medication-naïve patients and 86.4 weeks for medication-free patients with an average duration of 66.8 weeks of medication treatment in the van Eijndhoven *et al.* study.^19^ In addition, the studies differed in MR field strength, as van Eijndhoven *et al.* utilized a 1.5T MR scanner whereas we used a 3.0T scanner in the present study. Nevertheless, the observation of abnormalities in both the studies suggest a critical role of medial orbitofrontal cortex in the early developmental course of the depression, and the difference in direction of effect across studies suggest that the course of illness or treatment may significantly impact the integrity of this region.

According to recent human fMRI studies, medial orbitofrontal cortex contributes to the processing of reward and other hedonic qualities of life experience,^51^ and thus abnormalities in this region might be related to symptoms of anhedonia and apathy in MDD patients. Pars opercularis, another region with increased cortical thickness in our study, anatomically is in the ventrolateral prefrontal cortex and interacts with orbital prefrontal systems. Abnormalities in this region in depression are believed to be related to symptoms such as apathy, psychomotor slowing and impaired performance on tasks of attention and executive function.^52^ Rostral middle frontal gyrus, part of DLPFC, has an essential role in mood regulation, working memory and problem solving.^53^ Abnormalities of DLPFC have been broadly reported in post-mortem,^26^ neuroanatomical,^50^ functional neuroimaging^54, 55, 56^ and neuropsychological studies^57^ in MDD patients. Consistent with our study, increased cortical thickness in right rostral middle frontal gyrus was also observed by van Eijndhoven *et al.*^19^ in first-episode, medication-free MDD patients.

Medial orbitofrontal cortex and pars opercularis, which had increased cortical thickness in the present study, are part of medial prefrontal and orbital neural networks respectively. The rostral middle frontal gyrus shares many of its connections with the medial prefrontal network.^58^ Abnormalities in these two interconnected cortical circuits are believed to have critical roles in disturbances of affect regulation in patients with mood disorders.^58^ Considering our observation of greater cortical thickness in medial orbitofrontal cortex, pars opercularis and rostral middle frontal gyrus in first-episode, treatment-naïve, mid-life MDD patients, together with prior postmortem studies in MDD that have demonstrated modest thinning in orbitofrontal and dorsolateral prefrontal cortical regions,^26,27^ it may be that the cortices of these areas may have increased thickness during episodes of illness or close to illness onset whereas showing a declining thickness over the course of multiple episodes that could contribute to the persistent disability and reduced treatment response seen in some chronic patients. Such a process over the course of illness could contribute to the smaller orbitofrontal and dorsolateral prefrontal volume as seen in morphometric MRI studies in patients with recurrent MDD.^2, 3, 4,59^ Consistent with this possibility, a longitudinal VBM study found that gray matter reductions in DLPFC and medial orbitofrontal cortex progressively worsened in patients with chronic recurrent depression.^60^

Another interesting finding in the current study was the increased cortical thickness in right supramarginal gyrus which has been less commonly reported in MDD patients. Supramarginal and angular gyri constitute the inferior parietal lobule, which is involved in visual perception and visuomotor control. Previous studies suggest that these processes may be altered in depression,^61,62^ and that changes in the parietal lobe may contribute to some neuropsychological^63^ and sensorimotor coordination problems in MDD patients.^61^

The analysis of pial area revealed a trend toward increased surface area in the left parahippocampal gyrus. This is of interest because this region is an important source of input to the hippocampus, which, as many lines of work suggest, is impaired in depression. Although it is broadly consistent with cortical thickness measurements in showing increased rather than decreased regional brain size, it is notable for being in a different region from those showing alterations in cortical thickness (see Supplementary page 1). As these two morphometric parameters are essentially independent contributors to volume measurements and have different pathophysiological implications, this observation suggests that different pathophysiological processes may drive change in this region. This trend finding requires replication, as no surface area finding survived FDR correction for multiple comparisons. Overall, the pattern of findings in our study suggests that alterations in cortical thickness are more pronounced than those in pial area during the early stage of MDD. Secondary analyses revealed increased gray matter volume in the left paracentral lobule, left superior frontal gyrus, bilateral cuneus and thalamus, which indicate changes in regions different from those observed in cortical thickness and pial area analyses. Thus, although different regions were implicated when different measures were obtained, our multidimensional assessment of brain systems in first episode MDD provide consistent indication pointing toward increases rather than decreases in brain tissue.

Another interesting finding from the present study is the increased correlations of regional cortical thickness measurements across the brain in MDD patients relative to healthy controls. According to a recent study by He *et al.*,^32^ brain areas are often anatomically connected in relation to the correlations in their cortical thickness. Lerch *et al.*,^64^ taking Brodmann Area 44 as a seed region, reported patterns of anatomical correlations across the cerebral cortex that were strikingly similar to tractography maps obtained from diffusion tensor imaging. In contrast to the earlier observations of decreased functional connectivity within prefrontal-limbic-thalamic circuitry,^39^ present study showed increased correlations of cortical thickness across the regions with increased cortical thickness. Therefore, although the increased correlations of cortical thickness across brain regions in MDD patients may represent a consistent local effect across multiple cortical regions such as an inflammatory response, it is also possible that it may represent abnormal or compensatory cortico-cortical connections across multiple regions in parallel. In the latter case, the synchronous increases in cortical thickness across brain areas may reflect a close functional connection of the two networks whose alteration may be relevant for the pathogenesis of MDD. In either case, the enhanced correlations of changes in thickness of the right supramarginal gyrus with the three prefrontal regions in MDD patients suggest a wide scale process. Related findings from resting-state fMRI^65,66^ and diffusion tensor imaging^67^ studies also have reported altered neural circuitry/connectivity alterations in first-episode MDD patients, and these may be relevant for the mechanistic understanding of the cortical thickness findings of the present study.

Several potential limitations of this study should be noted. First, since our measurements of cortical thickness were restricted to the cortical mantle, we cannot assess the thickness of regions such as thalamus, hippocampus, amygdala, striatum, and accumbens, which are critical to MDD. Second, because this study was cross-sectional, our findings cannot address the question of whether the structural differences that we report close to illness onset reflect altered brain maturation that developed over time because of gene–environment interactions, cellular, molecular and epigenetic forms of plasticity over the years before illness onset, or pathophysiological changes associated with the acute onset of depression.^59^ Longitudinal clinical studies and studies of ‘at risk' individuals are needed to address these issues and questions about the longer-term course and clinical implications of our findings. Third, as the analyses of interregional anatomical connectivity were based on a correlational analysis of cortical thickness, it is not clear whether the altered correlations represent real alterations in functional connections or simply parallel effects of illness pathophysiology on different brain regions.

In conclusion, the present study complements and extends previous neuroimaging studies of MDD by demonstrating increased cortical thickness of prefrontal and parietal cortex in a large sample of first-episode, treatment-naïve, mid-life adult MDD patients mainly in regions where functional alterations have been demonstrated previously. As these findings of increased thickness vary notably from studies of chronic patients that suggest volume loss, future longitudinal imaging studies are needed to determine whether there are dynamic changes in cortical thickness, and regional changes of anatomical connection patterns and other aspects of brain anatomy over the course of MDD, to clarify the maturational and episode-related nature of these alterations of brain anatomy, their potential progression over the course of illness in MDD patients, and the potential impact of therapeutic intervention on these processes.

## Figures and Tables

**Table 1:** Demographic and clinical characteristics of first-episode, treatment-naive MDD patients and healthy controls

	*Mean (s.d.)*		
*Characteristic*	*First-episode, treatment-naive MDD patients (*N*=46)*	*Healthy controls (*N*=46)*	P*-value*
Male/female, no.	13/33	13/33	1.00
Age (years)	34.9 (10.8)	35.4 (11.5)	0.58
Education (years)	10.6 (3.2)	11.5 (2.9)	0.78
Illness duration (weeks)	17.4 (17.7)		
HDRS	23.3 (5.0)		

Abbreviations: HDRS, Hamilton depression rating scale; MDD, major depressive disorder.

**Table 2:** Location and clinical correlation of brain regions with altered cortical thickness in major depression

*Region*	*BA*	*Talairach*			*Size (mm*^*2*^)	−*log (*p*)*	*Correlation with Illness duration,* r *values*	*Correlation with HDRS,* r *values*
R-rostral middle frontal gyrus	46	33	56	−9	927	3.82	0.07 (*P*=0.652)	−0.34 (*P*=0.020)*
R-medial orbitofrontal gyrus	9,10	12	50	−1	1114	5.67	0.15 (*P*=0.332)	−0.11 (*P*=0.472)
R-supramarginal gyrus	40	51	−34	32	370	3.98	0.28 (*P*=0.059)	−0.29 (*P*=0.051)
R-pars opercularis	44	50	14	19	889	4.81	0.12 (*P*=0.443)	−0.07 (*P*=0.652)

Abbreviations: BA, Brodmann area; HDRS, Hamilton Depression Rating Scale; R, right. Coordinates of the minimum *P*-values in Talairach space are expressed as ‘x, y, z' in millimeters. *P<0.05 with two-tailed level.

**Table 3:** Correlation of cortical thickness measurements between brain regions with altered cortical thickness in MDD presented separately for MDD patients and healthy controls

	*MDD,* r	*HC,* r	Z
R-MOF~R-pars opercularis	0.50 (*P*=0.001*)	0.22 (*P*=0.142)	2.91*
R-MOF~R-RMF	0.54 (*P*<0.001*)	0.35 (*P*=0.019*)	2.26*
R-MOF~R-supramarginal gyrus	0.35 (*P*=0.018*)	0.22 (*P*=0.155)	1.39
R-pars opercularis~R-RMF	0.53 (*P*<0.001*)	0.26 (*P*=0.086)	3.05*
R-pars opercularis~R-supramarginal gyrus	0.41 (*P*=0.006*)	0.22 (*P*=0.154)	1.98*
R-RMF~R-supramarginal gyrus	0.32 (*P*=0.031*)	0.20 (*P*=0.193)	1.25

Abbreviations: HC, healthy control; MDD, major depressive disorder; MOF, medial orbitofrontal gyrus; R, right hemisphere; RMF, rostral middle frontal gyrus. *Z*-values reflect group differences in interregion association between MDD patients and healthy controls. *P<0.05 with two-tailed level.

## Notes

JAS received support from Roche, Takeda, BMS, and Lilly. The remaining authors declare no conflict of interest.
