# Supplementary material for: Regional increases of cortical thickness in untreated, first-episode major depressive disorder
Source: Transl Psychiatry. 2014 Apr 8;4(4):e378–. doi: 10.1038/tp.2014.18 (PMC4012282; doi:10.1038/tp.2014.18)
Supplement: Supplementary Information [file tp201418x1.doc]

**Supplementary Material**

***Regional increases of cortical thickness in untreated, first-episode major depressive disorder***

Lihua Qiu^＃^, Su Lui^＃^, Weihong Kuang, Xiaoqi Huang, Jing Li, Jin Li, Junran Zhang,

Huafu Chen, John A. Sweeney, and Qiyong Gong*

**Regional alterations of pial area in untreated, first-episode MDD**

Previous research suggests that surface area and cortical thickness reflect different neurobiological processes and are regulated by different genetic mechanisms.[^1^](#_ENREF_1) Analyzing cortical thickness and surface area separately may help to discriminate these two genetically independent traits. The radial unit hypothesis suggests that the cortical surface area is influenced by the number of columns, whereas cortical thickness is influenced by the number of cells within a column.[^2^](#_ENREF_2) To our knowledge, no study has yet investigated the changes of pial area (i.e., surface area) in MDD patients. Here, we present our preliminary findings of altered regional pial area in 46 untreated, first-episode patients with major depressive disorder compared with 46 age, sex and years of education matched healthy controls. We also explore the difference between the results of pial area and those of cortical thickness.

The construction of cortical surface was based on 3D SPGR images using FreeSurfer software (<http://surfer.nmr.mgh.harvard.edu/>, the vision of FreeSurfer used was 4.5.0). The differences of pial area between patients and controls were compared using a general linear model approach with age and sex as covariates. False discovery rate (FDR) correction (P<0.05) was performed for formal analysis providing full Type 1 error protection. In exploratory analysis, the statistical threshold for identifying effects of interest was set as p<0.001.

In exploratory analysis, only left parahippocampal gyrus (Talairach coordinates: -32, -42, -11; -log (p) = 3.12) showed increased pial area in MDD patients (p<0.001 uncorrected, Figure S1). This effect did not survive FDR correction (p<0.05). Our significant findings with cortical thickness measures suggest that the brain substrate of this measure is more significantly impaired at the early stage of MDD. In addition, it is noteworthy that the trend finding of pial area in the parahippocampal gyrus (notably an increase rather than decrease in pial area) was in an area where effects were not seen in cortical thickness studies, highlighting that surface area and cortical thickness measurements provide distinct information about cortical structure.


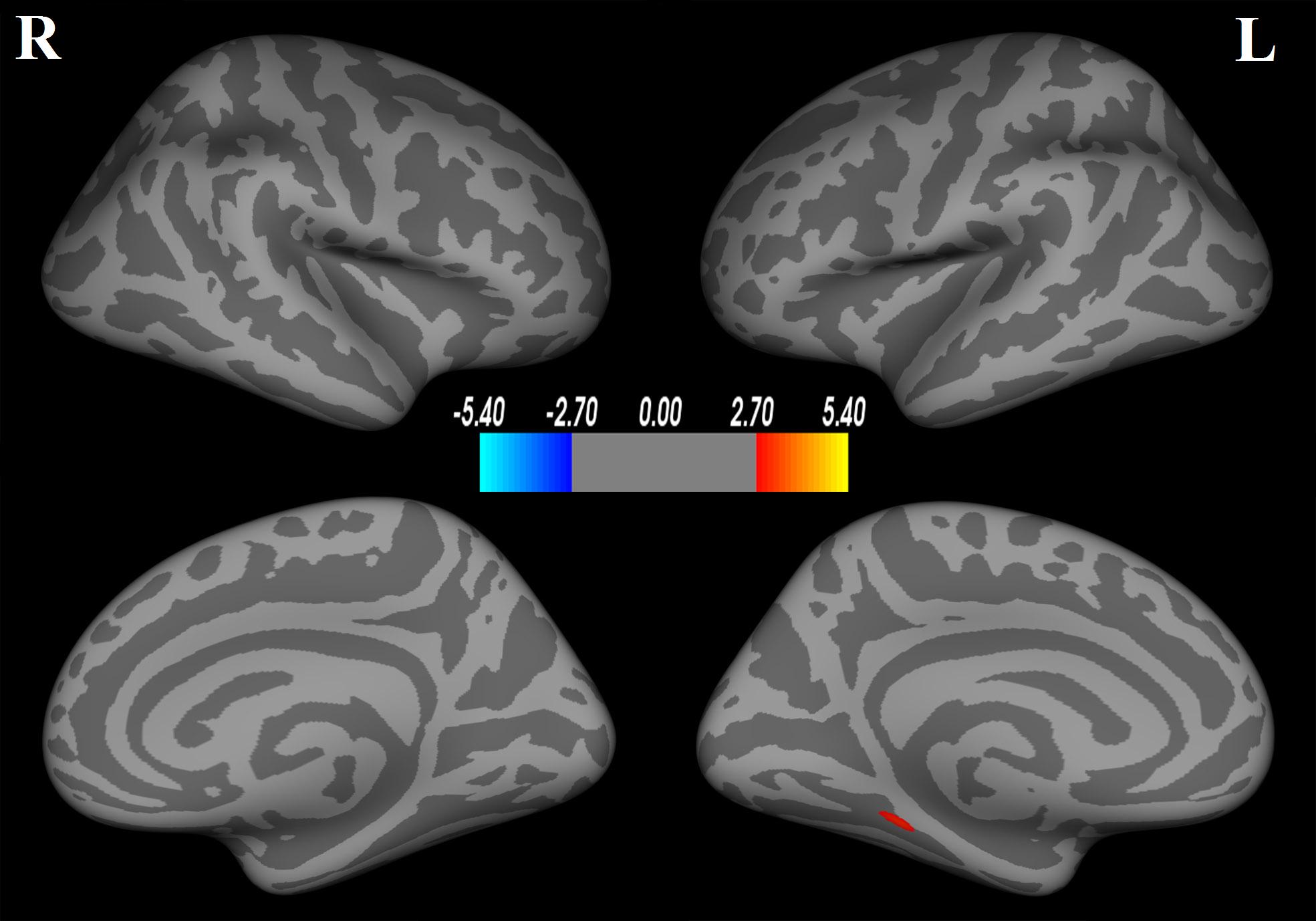


Figure S1: Compared to controls, MDD patients showed a trend (p<0.001) toward increased pial area in left parahippocampal gyrus. No area of decreased pial area was found in MDD patients. Warmer colors (positive values) represent increased pial area in MDD patients.

Previous studies have demonstrated that cortical areas participating in information-processing circuits through rich inter-areal connections and cortical networks are optimized to reduce wiring costs and save energy and time during signaling.[^3^](#_ENREF_3) So the regional altered surface area may influence the regional information-processing. A main function of parahippocampal gyrus involves memory processes and sending information from the hippocampus to association areas. Thus, the altered pial area of parahippocampal gyrus may be related with the impaired memory in MDD patients. A recent published study[^4^](#_ENREF_4) of first-episode, treatment-naive depression revealed lower regional homogeneity (ReHo) in left parahippocampal gyrus compared with controls, while another study found[^5^](#_ENREF_5) increased functional connectivity of left parahippocampal gyrus with right DLPFC in first-episode MDD patients. Our result extends previous fMRI studies by suggesting anatomic alteration in the parahippocampal gyrus in untreated, first-episode MDD patients.

Our result of increased pial area seemed contrary to previous reports of decreased parahippocampal gyrus volume in MDD. Although gray matter volume are generally more closely related to surface area than cortical thickness,[^8^](#_ENREF_8) the regional volume change might not be detected if the two morphometric parameters were altered in the inverse direction in the same region. Further, our patients were first-episode, untreated, mid-life MDD patients instead of chronic, medicated MDD patients. Thus, our result of the surface area cannot be compared with the volumetric results directly. In addition to possible inflammatory influences, the increased surface area in our present study may be due to the neuronal overgrowth or a deficit in the normal pruning process during neural maturation in MDD. The differential location of thickness and surface area abnormalities across the brain in MDD may be due to their different genetic bases and the different areas may be informative for different pathways of illness risk.

Taken together, present findings of altered pial area and cortical thickness are consistent with the view that these two morphometric parameters are essentially unrelated and thus can have different pathophysiological implications. Further, surface or thickness measures may have advantages over volume measures, and surface area and cortical thickness measurements should be considered separately.[^8^](#_ENREF_8)

**Volumetric changes in first-episode, untreated major depressive disorder: a VBM-DARTEL study**

Most previous morphologic studies of MDD that investigated volumetric changes used a voxel-based morphometry (VBM) analysis, and most of these studies included patients who were medicated or remitted chronic MDD patients. Inconsistent results have been found across those reports, including increased[^9-11^](#_ENREF_9) or reduced gray matter volume in hippocampus, anterior cingulate,[^14-16^](#_ENREF_14) orbitofrontal cortex, insula,[^18^](#_ENREF_18) dorsal anterolateral and ventrolateral prefrontal cortex, the precuneus and the inferior parietal lobule.[^16^](#_ENREF_16) Only two published studies in first-episode, drug-naïve MDD patients reported reduced volume in hippocampus[^19^](#_ENREF_19) and right pre-supplementary motor area[^20^](#_ENREF_20) using VBM analysis. Yet, investigating the large cohort first-episode, treatment-naïve MDD patients may help for elucidating the core pathophysiology of this illness. Therefore, in the present study, we also investigated volumetric change in first-episode, treatment-naive MDD patients using voxel-based morphometry.

All the 3D T1WI images of the 46 first-episode, treatment -naïve, mid-life MDD patients and 46 matched healthy controls were first converted to ANALYZE format and then were manually reoriented so that they were centered on the anterior commissure. VBM analysis was performed by using SPM8 software (http://www.fil.ion.ucl.ac.uk/spm), the VBM8 toolbox (<http://dbm.neuro.uni-jena.de/vbm8>). All images were segmented into gray matter (GM), white matter, and cerebrospinal fluid (CSF) and the segmentation procedure was further refined by high dimensional warping by applying a Diffeomorphic Anatomical Registration using Exponentiated Lie algebra (DARTEL) algorithm which is a more sophisticated registration algorithm and can be used to achieve a more accurate inter-subject registration. The resulting GM images were modulated to account for volume changes resulting from the normalization process. Non-linear GM volumes that were corrected for individual brain size were then smoothed with 8mm Full-Width at Half-Maximum (FWHM) smoothing kernel for final statistical analysis. Statistical group difference maps were constructed using a general linear model (GLM) with sex and age as covariates. The areas with p<0.05 after FDR correction with a minimum cluster size of 50 were considered significant.

Compared to controls, MDD patients demonstrated increased gray matter volume in the left paracentral lobule, left superior frontal gyrus, bilateral cuneus and bilateral thalamus. No significantly decreased gray matter volume was found in MDD patients. (Figure S2 and Table S1).


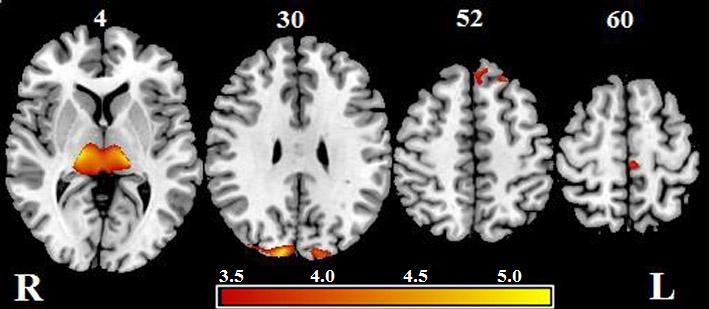


Figure S2: Compared to controls, the MDD patients demonstrated increased gray matter volume in the left paracentral lobule, left superior frontal gyrus, bilateral cuneus and bilateral thalamus. No decreased gray matter volume was found in MDD patients. Warmer colors (positive values) represent increased volume. Numbers above the brain images represent the vertical distance in millimeters from the anterior commissure.

**Table S1: Location of altered volume in major depressive d**isorder

| **Region** | **MNI** | | | **t** |
| --- | --- | --- | --- | --- |
|  | **X** | **Y** | **z** |  |
| **L-paracentral lobule** | -3 | -27 | 61 | 3.73 |
| **L- superior frontal gyrus** | -3 | 42 | 51 | 4.09 |
| **L-cuneus** | -12 | -91 | 28 | 4.01 |
| **L-thalamus** | -18 | -24 | 5 | 5.31 |
| **R-cuneus** | 12 | -90 | 30 | 5.15 |
| **R-thalamus** | 16 | -19 | 6 | 5.27 |

Abbreviations: L- left, R- right

The increased gray matter volume in cortical and subcortical regions is seemingly contradictory to the reported decreased gray matter volume in hippocampus and right pre-supplementary motor area in first-episode, drug-naïve MDD patients. However, the mean illness duration of the two studies (7.6 months and 10.98 months respectively) were much longer than the present study (17.4 weeks). In particular, it has been recognized that there was progressive volume loss over time in MDD patients.[^23^](#_ENREF_23) Notably, one of the above two studies comparing the first-episode MDD patients with short illness duration (illness duration<12 months) and long duration (illness duration between 12~24 months) also revealed greater whole gray matter volume and bilateral hippocampi volume in patients with short illness duration relative to patients with longer illness duration.[^20^](#_ENREF_20) Apart from the illness duration, it is also possible that first onset age and sample size may also influence the results.

As a primary sensorimotor cortex, the increased volume of left paracentral lobule may be related to the slowed and less accurate locomotor activity in depression. Altered volume of paracentral lobule had been reported in late-onset depression.[^24^](#_ENREF_24) Cuneus is an area involved in visual processing and imagery, the increased volume of cuneus may be related with the symptoms of repetitive processing of intrusive imagery involving past life stressors which common in patients with depression.[^25^](#_ENREF_25) Contrary to the increased gray matter volume, decreased gray matter density of cuneus has been found in depression patients.[^23^](#_ENREF_23) The different results may be due to the heterogeneity of patients and the different parameters for analysis. The volume change of superior frontal gyrus is the common findings in MDD. The medial superior frontal gyrus is part of the medial prefrontal network which can modulate visceral function in relation to emotion or other factors.[^28^](#_ENREF_28) Thalamus is an important output target of the limbic system, and thus plays key role in the expression and experience of emotion. The increased volume of bilateral thalamus is consistent with the elevated total number of neurons in the thalamus that have been reported in major depressive disorder.[^29^](#_ENREF_29) However, no decreased volumes were found in our present study seems contrary to published results with chronic patients. We speculate heterogeneity of patients, course of illness, medication, and comorbidity effects may explain the different results. All our patients were first-episode and treatment-naive, and thus the increased volume of these areas may represent pre-existing risk factors or effects of illness in the early stage of MDD. Similar results of increased gray matter volume and density also found in first-episode patients with bipolar disorder.[^30^](#_ENREF_30) Since our patients were first-episode depression, we can’t exclude the possibility of a future manic episode in our patients and thus that some of our patients may ultimately have bipolar disorder. Longitudinal study may help to clarify patients’ classification and dynamic change of these areas.

In summary, the present study confirmed volumetric changes in first-episode, treatment-naïve, mid-life MDD patients and these areas belong to limbic-cortico–striato–pallido–thalamic loops hypothesized to be central to MDD. These volumetric results could not be directly compared with our surface-based results due to different methodological factors that may influence the results.[^7^](#_ENREF_7) The voxel-based analytic method can’t detect variants of folding patterns and focal changes on grey matter volume, while it has the advantage for the analysis of gray matter in subcortical structures and for multivariate analysis that include voxel-based functional imaging.[^8^](#_ENREF_8) Furthermore, although volume measures parallel cortical thickness and pial area measures, the three morphologic parameters are believed to be genetically and phenotypically independent.[^8^](#_ENREF_8)

**References**

1. Panizzon MS, Fennema-Notestine C, Eyler LT, Jernigan TL, Prom-Wormley E, Neale M *et al.* Distinct genetic influences on cortical surface area and cortical thickness. *Cereb Cortex* 2009; **19**(11)**:** 2728-2735.

2. Rakic P. Specification of cerebral cortical areas. *Science* 1988; **241**(4862)**:** 170-176.

3. Im K, Lee JM, Lyttelton O, Kim SH, Evans AC, Kim SI. Brain size and cortical structure in the adult human brain. *Cereb Cortex* 2008; **18**(9)**:** 2181-2191.

4. Chen JD, Liu F, Xun GL, Chen HF, Hu MR, Guo XF *et al.* Early and late onset, first-episode, treatment-naive depression: same clinical symptoms, different regional neural activities. *J Affect Disord* 2012.

5. Ye T, Peng J, Nie B, Gao J, Liu J, Li Y *et al.* Altered functional connectivity of the dorsolateral prefrontal cortex in first-episode patients with major depressive disorder. *Eur J Radiol* 2012.

6. Peng J, Liu J, Nie B, Li Y, Shan B, Wang G *et al.* Cerebral and cerebellar gray matter reduction in first-episode patients with major depressive disorder: a voxel-based morphometry study. *Eur J Radiol* 2011; **80**(2)**:** 395-399.

7. Takao H, Abe O, Ohtomo K. Computational analysis of cerebral cortex. *Neuroradiology* 2010; **52**(8)**:** 691-698.

8. Winkler AM, Kochunov P, Blangero J, Almasy L, Zilles K, Fox PT *et al.* Cortical thickness or grey matter volume? The importance of selecting the phenotype for imaging genetics studies. *Neuroimage* 2009; **53**(3)**:** 1135-1146.

9. Leung KK, Lee TM, Wong MM, Li LS, Yip PS, Khong PL. Neural correlates of attention biases of people with major depressive disorder: a voxel-based morphometric study. *Psychol Med* 2009; **39**(7)**:** 1097-1106.

10. Scheuerecker J, Meisenzahl EM, Koutsouleris N, Roesner M, Schopf V, Linn J *et al.* Orbitofrontal volume reductions during emotion recognition in patients with major depression. *J Psychiatry Neurosci* 2010; **35**(5)**:** 311-320.

11. Yuan Y, Zhu W, Zhang Z, Bai F, Yu H, Shi Y *et al.* Regional gray matter changes are associated with cognitive deficits in remitted geriatric depression: an optimized voxel-based morphometry study. *Biol Psychiatry* 2008; **64**(6)**:** 541-544.

12. Bremner JD, Narayan M, Anderson ER, Staib LH, Miller HL, Charney DS. Hippocampal volume reduction in major depression. *Am J Psychiatry* 2000; **157**(1)**:** 115-118.

13. Sheline YI, Sanghavi M, Mintun MA, Gado MH. Depression duration but not age predicts hippocampal volume loss in medically healthy women with recurrent major depression. *J Neurosci* 1999; **19**(12)**:** 5034-5043.

14. Ballmaier M, Toga AW, Blanton RE, Sowell ER, Lavretsky H, Peterson J *et al.* Anterior cingulate, gyrus rectus, and orbitofrontal abnormalities in elderly depressed patients: an MRI-based parcellation of the prefrontal cortex. *Am J Psychiatry* 2004; **161**(1)**:** 99-108.

15. Caetano SC, Kaur S, Brambilla P, Nicoletti M, Hatch JP, Sassi RB *et al.* Smaller cingulate volumes in unipolar depressed patients. *Biol Psychiatry* 2006; **59**(8)**:** 702-706.

16. Salvadore G, Nugent AC, Lemaitre H, Luckenbaugh DA, Tinsley R, Cannon DM *et al.* Prefrontal cortical abnormalities in currently depressed versus currently remitted patients with major depressive disorder. *Neuroimage* 2010; **54**(4)**:** 2643-2651.

17. Lacerda AL, Keshavan MS, Hardan AY, Yorbik O, Brambilla P, Sassi RB *et al.* Anatomic evaluation of the orbitofrontal cortex in major depressive disorder. *Biol Psychiatry* 2004; **55**(4)**:** 353-358.

18. Takahashi T, Yucel M, Lorenzetti V, Tanino R, Whittle S, Suzuki M *et al.* Volumetric MRI study of the insular cortex in individuals with current and past major depression. *J Affect Disord* 2010; **121**(3)**:** 231-238.

19. Zou K, Deng W, Li T, Zhang B, Jiang L, Huang C *et al.* Changes of brain morphometry in first-episode, drug-naive, non-late-life adult patients with major depression: an optimized voxel-based morphometry study. *Biol Psychiatry* 2010; **67**(2)**:** 186-188.

20. Cheng YQ, Xu J, Chai P, Li HJ, Luo CR, Yang T *et al.* Brain volume alteration and the correlations with the clinical characteristics in drug-naive first-episode MDD patients: a voxel-based morphometry study. *Neurosci Lett* 2010; **480**(1)**:** 30-34.

21. Ashburner J, Friston KJ. Computing average shaped tissue probability templates. *Neuroimage* 2009; **45**(2)**:** 333-341.

22. Ashburner J. A fast diffeomorphic image registration algorithm. *Neuroimage* 2007; **38**(1)**:** 95-113.

23. Frodl TS, Koutsouleris N, Bottlender R, Born C, Jager M, Scupin I *et al.* Depression-related variation in brain morphology over 3 years: effects of stress? *Arch Gen Psychiatry* 2008; **65**(10)**:** 1156-1165.

24. Ballmaier M, Kumar A, Thompson PM, Narr KL, Lavretsky H, Estanol L *et al.* Localizing gray matter deficits in late-onset depression using computational cortical pattern matching methods. *American Journal of Psychiatry* 2004; **161**(11)**:** 2091.

25. Brewin CR, Lanius RA, Novac A, Schnyder U, Galea S. Reformulating PTSD for DSM-V: life after Criterion A. *J Trauma Stress* 2009; **22**(5)**:** 366-373.

26. Bilici MM, Ozalay O, Cagdas Eker M, Kitis O, Ozan E, Eker O *et al.* Small frontal gray matter volume in first-episode depression patients. *Turk Psikiyatri Derg* 2010; **21**(3)**:** 185-194.

27. Bremner JD, Vythilingam M, Vermetten E, Nazeer A, Adil J, Khan S *et al.* Reduced volume of orbitofrontal cortex in major depression. *Biol Psychiatry* 2002; **51**(4)**:** 273-279.

28. Price JL, Drevets WC. Neurocircuitry of mood disorders. *Neuropsychopharmacology* 2009; **35**(1)**:** 192-216.

29. Young KA, Holcomb LA, Yazdani U, Hicks PB, German DC. Elevated neuron number in the limbic thalamus in major depression. *Am J Psychiatry* 2004; **161**(7)**:** 1270-1277.

30. Adler CM, DelBello MP, Jarvis K, Levine A, Adams J, Strakowski SM. Voxel-based study of structural changes in first-episode patients with bipolar disorder. *Biol Psychiatry* 2007; **61**(6)**:** 776-781.
